# The Interface Between Parental and Occupational Burnout in Critical Care Unit Staff: A Multicentre Cross-Sectional Study

**DOI:** 10.3390/bs16101755

**Published:** 2026-09-26

**Authors:** Uxía Amoedo Fernández, Luis-Javier Márquez-Álvarez, Macarena Chacón-Docampo

**Affiliations:** 1 Research Unit in Care at the Vigo Health Area, SERGAS, 36212 Vigo, Spain; 2 Research Group in Care INVESTIC, Galicia Sur Health Research Institute (IIS Galicia Sur), SERGAS-UVIGO, 36312 Vigo, Spain; javier.marquez@udc.es; 3 Faculty of Health Sciences, University of A Coruña, 15006 A Coruña, Spain; 4 Escuela Universitaria Enfermería del Meixoeiro, Hospital do Meixoeiro, University of Vigo, 36214 Vigo, Spain

**Keywords:** professional burnout, parental burnout, critical care units, intensive care units, parenting, social support

## Abstract

Occupational burnout among Critical Care Unit (CCU) staff is a pressing issue, yet the relationship between parental burnout and this high-demand professional domain remains underexplored. Thus, this study aimed to analyze the association between parental and occupational burnout in critical care healthcare professionals. A multicentre cross-sectional study was conducted across three CCUs in hospitals within the Vigo Health Area (northern Spain). The sample included 106 CCU staff members with dependent children. Data were collected using the Burnout Screening Inventory, Parental Burnout Assessment, and Oslo Social Support Scale. Although the overall prevalence of occupational burnout was low in this sample, total parental burnout emerged as a statistically significant positive predictor of occupational burnout (β = 0.303, *p* = 0.003). Among its dimensions, parental emotional distancing acted as the most salient predictor (β = 0.409, *p* = 0.006). Parental burnout is significantly associated with higher levels of occupational burnout among CCU professionals, with parental emotional distancing being the most relevant dimension in this relationship, with perceived social support as an inverted association. These findings highlight the co-occurrence of both phenomena and the need to acknowledge the intersection of family demands and high-intensity work environments when addressing occupational wellbeing in critical care settings.

## 1. Introduction

Burnout has become one of the most pressing occupational health challenges confronting healthcare systems globally. Formally recognised by the World Health Organization (WHO) in 2019 as an occupational phenomenon within the International Classification of Diseases (ICD-11), burnout is defined as a syndrome arising from chronic workplace stress that has not been successfully managed (World Health Organization, n.d.). The tripartite model originally formulated by Maslach and Jackson conceptualises it through three interrelated dimensions: emotional exhaustion, depersonalisation (cynicism toward patients and colleagues), and a reduced sense of personal accomplishment (Edú-Valsania et al., 2022; Maslach & Jackson, 1981, 1982). Far from representing a transient state, burnout carries severe individual and organisational consequences, including deterioration of physical and mental health, increased absenteeism, voluntary turnover, and demonstrable reductions in the quality of patient care (Salvagioni et al., 2017).

Critical care unit (CCU) healthcare staff are exposed to a particularly demanding combination of working conditions that renders them among the most vulnerable occupational groups for burnout. The critical care setting is characterised by continuous monitoring and management of haemodynamically unstable patients, high clinical complexity, recurrent exposure to trauma and death, time-critical decision-making under uncertainty, moral distress, and chronic understaffing (Ballester Arnal et al., 2016; Gualano et al., 2021; Kok et al., 2023). These structural and emotional demands generate sustained cognitive and affective loads that habitually exceed available personal and organisational resources. Several systematic reviews and multicentre studies have documented substantial burnout rates in CCU staff. There are reports about pooled prevalence ranges from approximately 25% to over 50% depending on the instrument and population studied, with emotional exhaustion consistently identified as the most prevalent dimension (Gualano et al., 2021; Lin et al., 2025). The distinctive moral and emotional burden of critical care—including routine exposure to patient deaths, family suffering, and ethically complex situations—appears to amplify emotional exhaustion beyond the levels observed in less acuity-intensive hospital settings (Kok et al., 2023). These findings carry direct implications for patient safety, staff retention, and the long-term sustainability of critical care services.

In parallel with the study of occupational burnout, a conceptually distinct yet closely related construct has gained increasing empirical recognition: parental burnout. Mikolajczak and Roskam (Mikolajczak & Roskam, 2018) formalised this syndrome within their Balance between Risks and Resources (BR2) theoretical framework, defining parental burnout as a state of chronic exhaustion specifically arising from the demands of the parental role, characterised by emotional distancing from one’s children, a profound loss of parental efficacy, and a painful contrast between the parent one previously was and the parent one has become. Crucially, parental burnout is not a variant of occupational burnout, general fatigue, or clinical depression; it is a domain-specific syndrome with its own aetiology, measurement, and consequences (Mikolajczak & Roskam, 2018; Ren et al., 2024). Its global prevalence is substantial. A landmark transcultural study encompassing 17,409 parents across 42 countries identified individualistic cultural orientations and the “intensive parenting” norms they perpetuate as the dominant structural predictors of parental burnout, outweighing individual-level factors by a considerable margin (Roskam et al., 2021). In Spain, a latent-profile analysis using the validated Parental Burnout Assessment (PBA) estimated clinically significant parental burnout in 26.1% of the adult parent population, with approximately 6% exhibiting indicators severe enough to require psychiatric attention (Suárez et al., 2022).

The intersection of professional and parental demands in the lives of healthcare workers raises fundamental questions about how burnout experienced in the family domain may influence occupational functioning. Two major theoretical models provide the explanatory scaffolding for this relationship. The Job Demands–Resources (JD-R) Model proposes that burnout develops when work demands chronically exceed the personal and organisational resources available to manage them (Bakker et al., 2014, 2023). This framework has been extended to encompass family-domain demands; research in healthcare worker samples has shown that the combined burden of job and family demands is associated with burnout above and beyond either domain alone (Maglalang et al., 2021). Concurrently, Hobfoll’s Conservation of Resources (COR) Theory posits that individuals possess a finite reservoir of physical, emotional, and cognitive resources, and that burnout is triggered when those resources are threatened, lost, or fail to generate expected returns (Hobfoll, 1989); individuals already depleted in one domain carry heightened vulnerability to resource loss in others, placing parents experiencing burnout at home at elevated risk for occupational exhaustion. These processes may operate through the spillover–crossover framework, which describes how emotions, attitudes, and behavioural states transfer within a person across life domains (spillover) and propagate interpersonally to others in shared environments (crossover) (Ratnaningsih & Idris, 2024; Tsukerman et al., 2020). Work–family conflict—defined as the inter-role incompatibility arising when competing demands from both domains become irreconcilable—has been consistently associated with elevated burnout, reduced job satisfaction, and increased absenteeism across healthcare populations (Cañadas-De la Fuente et al., 2018; Tsukerman et al., 2020).

The critical care unit context presents a particularly challenging configuration for the parental–occupational burnout interface. Rotating shift schedules, characteristic of critical care services, structurally disrupt parenting routines and co-parenting coordination that ordinarily buffer parental burnout (Roskam et al., 2021). Unlike settings where professional demands are more predictable, the critical care environment requires continuous emotional and cognitive availability across each shift—a demand that individuals already experiencing parental role exhaustion may be particularly ill-equipped to sustain (Evans et al., 2023; Mikolajczak & Roskam, 2018). From a COR perspective, CCU professionals who enter their shifts with pre-existing parental resource deficits face an elevated risk of accelerated resource depletion; subsequent high-impact events—patient deaths, family crises, or situations of ethical conflict—may compound rather than merely add to that burden (Hobfoll, 1989; Kok et al., 2023). Limited scheduling flexibility, frequent overtime, and chronic staffing shortages further restrict the organisational buffering mechanisms that might otherwise interrupt this dynamic, making the critical care unit a context in which the parental–occupational burnout interface may carry amplified consequences.

A growing body of longitudinal evidence has begun to clarify the specific directional relationship between parental burnout and occupational burnout. Cross-lagged panel model analyses have consistently identified parental burnout as a significant predictor of subsequent occupational burnout, while the reverse pathway has not reached statistical significance (Wang et al., 2022, 2023). Wang et al. (2023) demonstrated in a three-wave longitudinal study that prior parental burnout robustly predicted later job burnout, whereas prior job burnout exerted no significant influence on later parental burnout. A subsequent actor–partner interdependence model study of dual-earner couples confirmed not only that parental burnout spills over into one’s own job burnout via work–family conflict, but also that cross-partner effects are statistically significant—one partner’s parental exhaustion measurably increasing the other’s occupational depletion (Wang et al., 2022). Evans et al. (2023) further demonstrated that parental burnout exerts both direct and indirect effects on work-related outcomes, including emotional exhaustion and professional disengagement, through mechanisms that are not reducible to shared dispositional or contextual factors. Mechanistically, the profound feelings of failure, guilt, hypervigilance, and hypothalamic–pituitary–adrenal axis dysregulation that accompany parental burnout may compromise executive function and emotional regulation in ways that are not domain-specific. Individuals experiencing parental burnout may arrive at work already cognitively and emotionally depleted (Evans et al., 2023; Mikolajczak & Roskam, 2018), a circumstance that, in the high-acuity critical care setting, may carry particularly consequential implications for professional performance and patient safety.

Despite the accumulating theoretical and empirical basis for this relationship, research specifically examining the role of parental burnout in occupational burnout among critical care unit staff remains scarce. Most available evidence derives from general working-population samples or from healthcare settings with different demand profiles, leaving a substantial gap in understanding this relationship within the critical care unit—a professional environment with its own distinctive stressor constellation, shift structures, and emotional burden (Gualano et al., 2021; Lin et al., 2025). Furthermore, the preponderance of existing studies relies on single-centre convenience samples, limiting the generalisability of findings across diverse institutional and clinical contexts.

The aim of this study is to examine the association between parental and occupational burnout, as well as the factors that may be related to this association in the field of critical care. To date, no study has examined parental burnout and occupational burnout simultaneously in a sample of critical care unit staff.

## 2. Materials and Methods

### 2.1. Study Design

A cross-sectional, descriptive–correlational, multicentre study was conducted. The cross-sectional design was selected as appropriate for estimating the prevalence and co-occurrence of parental and occupational burnout and for examining their association and its predictors at a single point in time. The study is reported in accordance with the Strengthening the Reporting of Observational Studies in Epidemiology (STROBE) guidelines for cross-sectional studies (Cuschieri, 2019).

### 2.2. Setting

The study was carried out in three CCUs at different hospitals within the Vigo Health Area, in Galicia (northern Spain). These departments have separate areas organised according to patient type, comprising a post-surgical unit for care lasting more than 48 h, a unit specialising in neurology and a unit specialising in cardiology. The average length of stay for patients in each unit varies considerably, ranging from short-term admissions (minimum of 48 h) to prolonged stays of up to 3 months.

The direct care team consists of physicians, nurses and nursing assistants. Care provision is organised using a hybrid shift model combining two rotation systems: one comprising 7-7-10-h morning, afternoon and night shifts, and the other comprising 12-h day and night shifts.

Preparatory activities, including liaising with the units and obtaining authorisation, were carried out in January 2026. Data collection took place between February and July 2026, following approval from the Institutional Review Board of the Nursing Management of the Vigo Health Area.

### 2.3. Participants

#### 2.3.1. Eligibility Criteria

The target population comprised healthcare professionals actively deployed in the CCU of the participating hospitals. The eligible professional categories were physicians, registered nurses, nursing assistants, and healthcare orderlies. No minimum duration of professional experience or department seniority was required as an eligibility criterion; seniority in the unit was captured as a descriptive variable.

The following inclusion criteria were specified:

- Active healthcare professional currently assigned to an CCU at a participating centre, in any of the eligible professional categories.
- At least one cohabiting dependent child at the time of data collection.
- Voluntary informed agreement to participate.

The following were included as exclusion criteria:

- Staff dedicated exclusively to administrative or management functions at the time of data collection.
- Professionals on medical or caregiving leave during the data collection period.
- Professionals undergoing active separation or divorce proceedings at the time of the study.

#### 2.3.2. Sampling Approach and Data Collection Procedure

Participants were recruited using a non-probabilistic convenience sampling approach combined with snowball sampling. Unit supervisors and nursing coordinators at each participating centre acted as primary contacts, distributing a questionnaire link to eligible staff via institutional communication channels and in daily team briefings. Participants were invited to forward the survey link to colleagues who met the inclusion criteria.

The complete instrument battery was administered as a self-completed electronic questionnaire hosted on Google Forms. An information letter presented at the start of the survey detailed the study objectives, the voluntary nature of participation, the right to withdraw without consequence at any time, and the anonymity and data protection measures in place. No personal identifiers were collected. All responses were stored in anonymised, password-protected files accessible exclusively to the research team.

Although physicians were among the eligible professional categories, no physicians consented to participate. The final sample therefore comprised registered nurses, nursing assistants, and healthcare orderlies. This constitutes a limitation discussed.

With respect to the parental-role composition of the sample, cohabitation status was recorded as a sociodemographic variable. All 106 participants reported cohabiting with a partner or co-parent (100%). The absence of single parents may reflect the true composition of the eligible population within the participating CCU, or alternatively a self-selection effect whereby single parents—facing greater logistical and temporal barriers—were less likely to consent to participation. This uncertainty is acknowledged as a potential source of bias and as a limitation of the study.

### 2.4. Variables

Occupational burnout was the primary outcome, operationalised as the total score on the Burnout Screening Inventory (BSI) (Dávila & Nevado, 2016). A categorical burnout severity classification (unlikely/mild/moderate) derived from validated BSI cut-off criteria was used for descriptive and chi-squared analyses.

Parental burnout, operationalised as the total score on the Parental Burnout Assessment (PBA) (Roskam et al., 2018) and its four subscales (parental emotional exhaustion, contrast with previous parental self, feelings of being fed up, and emotional distancing from one’s children), was defined as the primary predictor of occupational burnout.

Three variables were pre-specified a priori as covariates in the regression models, based on established theoretical frameworks and prior empirical evidence.

Perceived social support and workplace flexibility were selected on the basis of Hobfoll’s Conservation of Resources (COR) theory (Hobfoll, 1989) and Job Demands–Resources (JD-R) (Bakker et al., 2014) framework, which identify social support and organisational resources as protective buffers against the spillover of demands between work and personal life. In different settings specifically, the structural inflexibility imposed by rotating shifts and mandatory overtime has been identified as an amplifier of work–family conflict (Ballester Arnal et al., 2016; Kok et al., 2023), making perceived flexibility a theoretically justified contextual resource to control for. In the regression models, flexibility and social support scores were entered as pre-specified covariates, not as a primary explanatory variable.

Sex was included as a covariate given documented sex differences in burnout profiles across healthcare populations (Alanazy & Alruwaili, 2023; Kok et al., 2023).

Finally, the following variables were recorded for sample characterisation: age, professional category, years of professional experience, type of employment contract, seniority in the unit, number of cohabiting dependent children, and cohabitation status.

### 2.5. Data Sources

The following validated instruments and purpose-designed questionnaire were administered as part of the survey battery. According to local regulations, no personal identifiers were collected. All responses were stored in anonymised, password-protected files accessible exclusively to the research team.

#### 2.5.1. Sociodemographic Questionnaire

A purpose-designed questionnaire captured the following sociodemographic and occupational variables.

- Personal and demographic variables: sex (binary: male/female); age (open-ended integer, subsequently grouped as 30–40, 40–50, >50 years).
- Occupational variables: professional category (four options: physician, registered nurse, nursing assistant, healthcare orderly); type of employment contract (binary: permanent/temporary); years of professional experience (continuous, open-ended integer); seniority in the unit (continuous, subsequently grouped as 0–2, 2–5, 5–10, >10 years).
- Parenting and family responsibility variables: Two items specifically characterised the parental status and family configuration of participants, given the study’s focus on the parental–occupational burnout interface: (1) number of cohabiting dependent children (open-ended integer response); (2) cohabitation status (binary: co-parenting partner present vs. single parent). No validated parenting-responsibility scale was included in the sociodemographic module; the parental-role burden construct was operationalised through the Parental Burnout Assessment, while the family configuration variables served as descriptive characterisation of the sample.

#### 2.5.2. Burnout Screening Inventory (BSI)

Occupational burnout was measured using the Spanish-validated BSI (Dávila & Nevado, 2016), a 10-item instrument evaluating three core burnout dimensions: Emotional Exhaustion (BSI-EE; 4 items), Depersonalisation (BSI-DP; 3 items), and Reduced Professional Efficacy (BSI-PA; 3 items). Items are rated on a 7-point Likert-type frequency scale (0 = never; 6 = every day); the total score ranges from 0 to 60, with higher scores indicating greater severity. Validated categorical cut-off criteria allow classification into burnout severity groups (<30: Unlikely; 30–39: Mild; 40–49: Moderate; >50: Severe).

The BSI was selected over the Maslach Burnout Inventory—Human Services Survey (MBI-HSS) for its reduced participant burden (10 vs. 22 items), its validation in Spanish health-related personnel, its response-scale compatibility with the PBA (both use the same 0–6 Likert format), and its provision of validated categorical cut-off scores (Dávila & Nevado, 2016).

In the Spanish validation study, overall internal consistency was adequate (α = 0.71) (Dávila & Nevado, 2016). In the present sample, overall internal consistency was acceptable (α = 0.730; ω = 0.729). Subscale-level internal consistency in the present sample was as follows: BSI-EE ω = 0.638; BSI-DP ω = 0.614; BSI-PA ω = 0.717.

#### 2.5.3. Parental Burnout Assessment (PBA)

Parental burnout was assessed using the PBA (Roskam et al., 2018), a 23-item instrument rated on a 7-point Likert-type frequency scale (0 = never; 6 = every day), structured across four dimensions: parental emotional exhaustion (PBA-EE; 9 items), contrast with previous parental self (PBA-Contrast; 6 items), feelings of being fed up with the parental role (PBA-Fed up; 5 items), and emotional distancing from one’s children (PBA-Distancing; 3 items) (Roskam et al., 2018). Higher scores indicate greater parental burnout.

The Spanish validation by Suárez et al. (2022) confirmed the four-factor structure and demonstrated excellent internal consistency across all subscales (McDonald’s ω = 0.93–0.97). In the present sample, overall internal consistency was ω = 0.933. Subscale-level internal consistency in the present sample was as follows: PBA-EE ω = 0.879; PBA-Contrast ω = 0.900; PBA-Fed up ω = 0.652; PBA-Distancing ω = 0.658.

#### 2.5.4. Oslo Social Support Scale (OSSS-3)

Perceived social support was measured using the OSSS-3 (Kocalevent et al., 2018), a three-item instrument yielding a total score ranging from 3 to 14. Respondents are classified as having poor (3–8), moderate (9–11), or strong (12–14) perceived social support. The OSSS-3 has been standardised for general-population use and demonstrates adequate criterion validity across diverse populations (Kocalevent et al., 2018). This instrument was selected a priori to operationalise perceived social support as a contextual resource within the Conservation of Resources and JD-R framework (Bakker et al., 2014; Hobfoll, 1989).

Because the OSSS-3 comprises three conceptually distinct facets of social support (network size, perceived interest of others, and availability of practical help), it constitutes a composite rather than a unidimensional reflective scale; internal consistency indices such as ω are therefore not the primary validity criterion (Bollen & Diamantopoulos, 2017). Consistent with this, the McDonald’s ω obtained in the present sample was 0.584, a value within the range commonly reported for this instrument in heterogeneous occupational samples—and does not preclude its use as a composite score.

#### 2.5.5. Perceived Workplace Flexibility

Perceived workplace flexibility was assessed with a single item rated on a Likert-type scale from 1 (not at all flexible) to 5 (completely flexible), capturing the participant’s perception of how well their working schedule accommodates their parental responsibilities. This item was included a priori based on the Job Demands–Resources (JD-R) model (Bakker et al., 2014, 2023), which identifies schedule flexibility as an organisational resource buffering the spillover of parental demands into occupational functioning.

### 2.6. Bias

The use of non-probabilistic convenience and snowball sampling limits the representativeness of the sample and precludes random selection from a defined sampling frame. To mitigate this, data were collected across multiple CCU of multiple hospitals, enhancing external validity relative to single-institution designs. However, it is not possible to characterise the sample from each CCU, as this data was not collected in order to preserve the anonymity of the participants, given that it could lead to their identification. Therefore, no response rate could be calculated.

Participation was voluntary, and no data are available on non-participants. Two specific patterns of potential non-response merit explicit acknowledgement. First, although physicians were included among the eligible professional categories, none consented to participate; this professional group may systematically differ from nursing staff in burnout exposure or parental-role characteristics, and conclusions should not be extrapolated to CCU physicians. Second, all participating parents reported cohabiting with a partner or co-parent, with no single parents in the sample. Whether this reflects the true composition of the CCU workforce or the under-recruitment of a more logistically constrained subgroup cannot be determined from the available data. Both patterns are acknowledged as limitations.

All variables, including the outcome, main predictor, and covariates, were collected via self-report in the same survey session. This introduces the risk of common-method bias inflating observed associations. Social desirability effects may have additionally attenuated burnout disclosure, particularly among participants concerned about professional repercussions. No procedural remedy was available given the study design; this is acknowledged as an inherent limitation of self-report cross-sectional designs.

Finally, professionals experiencing severe burnout may have voluntarily transferred out of the CCU setting or left clinical practice prior to recruitment, resulting in a sample that underrepresents the most severely affected individuals. This may partially account for the low overall burnout prevalence observed and should be considered when interpreting the findings.

### 2.7. Study Size

Sample size was estimated a priori using G*Power 3.1 software (Faul et al., 2007). Based on conservative estimates from the work–family spillover literature, a minimum detectable effect of ρ = 0.30 was assumed for the bivariate relationship between parental burnout and occupational burnout. For a two-tailed correlation test at α = 0.05 and statistical power of 80% (1 − β = 0.80), the minimum required sample was *n* = 82. Recruitment continued across all participating centres until this threshold was reached or exceeded; a final sample of *n* = 106 was obtained, representing approximately a 29% above the minimum requirement.

### 2.8. Quantitative Variables

Continuous scores on the BSI, PBA, and OSSS-3 were retained as continuous variables in all correlation and regression analyses, as this preserves statistical power and avoids the information loss inherent in arbitrary categorisation.

The BSI total score was additionally categorised into three severity groups (unlikely burnout/mild burnout/moderate burnout) using the validated cut-off criteria from the Spanish normative study (Dávila & Nevado, 2016). These categories were used solely for descriptive purposes and for chi-squared analyses examining associations between burnout severity and categorical sociodemographic variables; they were not used in regression modelling.

The OSSS-3 total score was similarly categorised into poor, moderate, and strong support for descriptive presentation in Table 1, while the continuous score was used in regression and correlation analyses.

Age and seniority in the unit were grouped into theoretically meaningful categories for sample characterisation in Table 1 but were not entered as predictors in the regression models due to the sample size constraints.

### 2.9. Statistical Methods

All statistical analyses were performed in R (version 4.5.1). The significance threshold was set at α = 0.05 for all inferential tests.

Frequencies and percentages were reported for categorical variables. Means, standard deviations, medians, and interquartile ranges were reported for continuous and ordinal variables. The distributional properties of continuous variables were evaluated using the Shapiro–Wilk test. Given confirmed non-normality across all burnout and covariate scores (all Shapiro–Wilk *p* < 0.05), non-parametric procedures were applied throughout.

Associations between continuous variables were examined using Spearman’s rank-order correlation coefficient (ρ). Between-group comparisons for two independent groups were conducted using the Mann–Whitney U test; comparisons involving three or more groups were conducted using the Kruskal–Wallis H test, with post hoc pairwise contrasts applying Dunn’s procedure with Bonferroni correction. Associations between categorical burnout severity and categorical sociodemographic variables were examined using Pearson’s chi-squared test.

To examine the predictive contribution of parental burnout to occupational burnout, two complementary linear regression models were estimated with BSI total score as the outcome variable. Model 1 included the PBA total score alongside the pre-specified covariates (perceived social support, work schedule flexibility, sex, and number of children), allowing estimation of the parental–occupational burnout association while controlling for relevant contextual variables. Model 2 included the four PBA subscales (PBA-EE, PBA-Contrast, PBA-Fed up, PBA-Distancing) as predictors, with the aim of identifying which specific dimension of parental burnout carried the greatest independent predictive weight on occupational burnout. The normality of regression residuals was evaluated using the Shapiro–Wilk test; given confirmed non-normality, the robustness of all regression estimates was verified using bootstrap resampling with 5000 replications.

No item-level or participant-level missing data were identified in the dataset; all 106 participants provided complete responses across all instruments. Accordingly, no imputation procedures were applied.

### 2.10. Ethical Considerations

The study was conducted in full accordance with the ethical principles of the Declaration of Helsinki and applicable Spanish and European data protection legislation (Ley Orgánica 3/2018; Regulation EU 2016/679, GDPR). Ethical approval was granted by the Institutional Review Board of the Nursing Management of the Vigo Health Area (approval code: 130-2-26). Informed consent was obtained from all participants through voluntary completion and submission of the questionnaire, preceded by an information letter detailing the study purpose, voluntary nature of participation, anonymity guarantees, and the right to withdraw at any time without consequence. No personal identifiers were collected; all data were stored in anonymised, password-protected files and used solely for the purposes described in this study.

## 3. Results

### 3.1. Descriptive Analysis

In total, *n* = 106 healthcare professionals from the CCUs took part in this study. The sample was predominantly female (*n* = 83; 78.3%), with the majority of participants in the 40–50 age group (*n* = 56; 52.8%). Of the participants, 71.7% were on permanent contracts (*n* = 76) and 61.3% were qualified nurses (*n* = 65). More than three-quarters had been working in the unit for five years or more (*n* = 81; 76.4%). The full sociodemographic characteristics are presented in Table 1.

With regard to the categorical distribution of occupational burnout, 92.5% of participants were classified in the ‘unlikely’ category, 6.6% exhibited mild burnout and 0.9% moderate burnout. With regard to perceived social support, 46.2% reported poor social support and 44.3% reported moderate social support, whilst only 9.4% of participants reported high social support.

Shapiro–Wilk tests confirmed significant deviations from normality in all continuous variables (all *p* < 0.05), which justified the use of non-parametric statistics in the correlational analyses (Table 2).

The mean levels of occupational burnout measured using the BSI were relatively low in the sample. The emotional exhaustion subscale yielded the highest scores (BSI-EE: Me = 6.00; M = 6.36; SD = 3.97), followed by reduced personal fulfilment (BSI-PA: Me = 6.00; M = 6.65; SD = 3.67) and depersonalisation (BSI-DP: Me = 2.00; M = 3.07; SD = 3.44).

Parental burnout showed greater variability. The total PBA score showed moderate medians and means (median = 25.00, IQR = 27.75) and a mean of 26.59 (SD = 17.13). The parental emotional exhaustion subscale had the highest scores (PBA-EE: Me = 13.00; M = 14.83; SD = 8.23), whilst emotional distancing had the lowest scores (PBA-Distancing: Me = 3.00; M = 2.61; SD = 2.59). The full descriptive statistics are presented in Table 2.

The chi-squared analysis revealed no statistically significant associations between the category of work-related burnout and any sociodemographic variable: gender (χ^2^(1) = 0.263, *p* = 0.608), age group (χ^2^(2) = 3.427, *p* = 0.180), type of contract (χ^2^ (1) = 0.000, *p* = 1.000), perceived social support (χ^2^(2) = 2.238, *p* = 0.327) or length of service (χ^2^(3) = 1.266, *p* = 0.737). For these analyses, the sole case categorized as ‘Moderate’ had to be excluded. Similarly, in the case of the ‘professional’ category, the number of values (*n* < 5) in some cells did not allow for reliable results; therefore, these were not included in the reported results.

Furthermore, in the comparison of total scores, the analysis of the depersonalization subscale (BSI-DP) scores by gender using the Mann–Whitney U test revealed a significant effect (U = 1282.50; *p* = 0.003). Men had significantly higher average ranges compared with women (average range for men = 69.80 vs. average range for women = 48.55). When assessing the magnitude of this difference, a biserial rank correlation of r_B_ = 0.41 (95% CI [0.16, 0.61]) was obtained, indicating a moderate-to-large effect size. No significant differences by sex were detected in the other dimensions of occupational burnout.

### 3.2. Correlational Analysis

Spearman’s correlations were calculated between the PBA subscales, the BSI subscales and the main covariates (Table 3). As the distribution of all variables deviated from normality, this non-parametric method was the appropriate statistical procedure.

The four subscales of the PBA showed positive and significant associations with the BSI-EE and the BSI-PA. The parental emotional distancing subscale showed the strongest correlations with both dimensions of occupational burnout (PBA-Distancing × BSI-EE: ρ = 0.376, *p* < 0.001; PBA-Distancing × BSI-PA: ρ = 0.424, *p* < 0.001). No PBA subscale was significantly associated with depersonalisation at work (BSI-DP; all *p* > 0.05), which might suggest that parental burnout is specifically related to the emotional and performance components of occupational burnout, but not to emotional detachment or indifference towards work.

With regard to the support variables relating to structural and personal resources that may influence burnout, the role of social support and work flexibility was analysed. Perceived social support (OSSS-3) showed significant negative correlations with emotional exhaustion (ρ = −0.251, *p* = 0.009) and reduced personal fulfilment (ρ = −0.254, *p* = 0.009), indicating a protective role against workplace burnout. Interestingly, it also correlated positively with the number of children (ρ = 0.262, *p* = 0.007).

Furthermore, flexibility was negatively associated with emotional exhaustion at work (ρ = −0.193, *p* = 0.047) and with three subscales of parental burnout (PBA-EE: ρ = −0.239, *p* = 0.013; PBA-Contrast: ρ = −0.357, *p* < 0.001; PBA-Distancing: ρ = −0.235, *p* = 0.015), once again suggesting that this resource may act as a protective factor.

### 3.3. Regression Models

#### 3.3.1. Model 1: Total Parental Burnout as a Predictor of Occupational Burnout

A multiple linear regression model was estimated with the total BSI score as the dependent variable. The total PBA score was included as a predictor, together with the study’s independent variables: gender, number of children, perceived social support (OSSS-3) and perceived work flexibility (Table 4). As the Shapiro–Wilk test ruled out normality of the residuals in all preliminary models, bootstrapping (5000 replications) was applied to estimate robust confidence intervals and confirm the statistical significance of the coefficients.

The model as a whole proved to be statistically significant (F(5, 100) = 3.326, *p* = 0.008) and explained 14.4% of the variance in work-related burnout (R^2^ = 0.144; adjusted R^2^ = 0.101). The total parental burnout score was a positive and significant predictor of occupational burnout (*B* = 0.136, β = 0.303, *p* = 0.003), such that higher levels of parental burnout were associated with higher scores on the occupational burnout scale. It was also found that perceived social support, as measured by the OSSS-3, had an inverted association, emerging as a significant negative predictor of total BSI scores (*B* = −0.816, β = −0.211, *p* = 0.032).

#### 3.3.2. Model 2: Sub-Scales of Parental Burnout as Predictors of Occupational Burnout

With the aim of identifying which specific dimension of parental burnout has the greatest predictive power over occupational burnout, a second multiple linear regression model was estimated, with the four subscales of the PBA as predictors and the total BSI score as the dependent variable (Table 5).

The model was statistically significant (F(4, 101) = 4.640, *p* = 0.002) and explained 15.7% of the variance (R^2^ = 0.157, Adjusted R^2^ = 0.123). Among the subscales, parental emotional distancing (PBA-Distancing) was the only predictor to reach statistical significance (*B* = 1.215, β = 0.409, *p* = 0.006), indicating that the tendency to distance oneself emotionally from one’s children is the dimension of parental burnout with the greatest predictive power regarding occupational burnout. The remaining subscales (PBA-EE, PBA-Contrast, PBA-Fed up) did not contribute independently to the model in this sample. An analysis to assess the collinearity of the subscales was completed to examine its effects on the results. Multicollinearity was not a significant concern, as VIF and Tolerance values were within acceptable limits, though the theoretical overlap between subscales suggests individual predictors should still be interpreted cautiously.

## 4. Discussion

The present multicentre study investigated the relationship between parental burnout and occupational burnout in CCU healthcare professionals. The results show that parental burnout was statistically significantly and positively associated with occupational burnout (R = 0.379, R^2^ = 0.144, *p* = 0.008), with parental emotional exhaustion as the dimension showing the strongest association in the subscale analysis. Taken together, the results are consistent with the predictions of Conservation of Resources (COR) theory (Hobfoll, 1989) and the spillover–crossover model (Ratnaningsih & Idris, 2024; Tsukerman et al., 2020) and extend the emerging evidence base on the parental–occupational burnout interface to the context of CCU work.

### 4.1. Parental Burnout of the Sample

The median PBA score was 25.00 (mean = 26.59, SD = 17.13), indicating moderate average parental burnout in this sample. To provide an appropriate context, Suárez et al. (2022) estimated that approximately 26.1% of the adult Spanish parent population presented clinically significant parental burnout, with about 6% requiring psychiatric attention. The data observed here fall within a comparable range. This finding is significant because it describes the sample’s profile beyond the workplace. Apparently, the CCU professionals who participated in the study simultaneously face clinically significant parental burdens and high-intensity work demands. This is a combination that theoretical frameworks identify as a vulnerability factor (Bakker et al., 2014; Hobfoll, 1989; Mikolajczak & Roskam, 2018).

Among the dimensions of the PBA, parental emotional exhaustion scored highest (median = 13.00; M = 14.83; SD = 8.23). This could be referred to the central role of emotional exhaustion described in the BR2 framework by Mikolajczak and Roskam (Mikolajczak & Roskam, 2018) and corroborated in international normative studies (Roskam et al., 2018; Suárez et al., 2022). This pattern is theoretically consistent because emotional exhaustion constitutes the primary and initiating dimension of parental burnout syndrome, from which emotional detachment and parental saturation develop sequentially (Bakker et al., 2014; Mikolajczak & Roskam, 2018). Parental emotional distancing (median = 3.00) and parental saturation (median = 3.00) were the least expressed dimensions, suggesting that parental burnout in this sample manifests primarily as emotional fatigue rather than as a disconnection from the parental role. From a clinical perspective hypothesis, this profile could indicate that the participants were in an early or middle stage of the parental burnout process, in which chronic fatigue has not yet led to sustained emotional detachment from their children.

### 4.2. The Interface Between Parental and Occupational Burnout in CCU

The central finding of this study, that PBA is positively and statistically correlated with BSI scores (β = 0.303, F(5, 100) = 3.326, *p* = 0.008, R^2^ = 0.144), is consistent with the longitudinal and cross-sectional literature on the relationship between parental and occupational burnout. Wang et al. (2023) demonstrated in a three-wave cross-lagged panel design that parental burnout robustly predicted subsequent occupational burnout, while the reverse pathway did not reach significance. This pattern of asymmetry suggests that transmission between domains occurs predominantly from the family sphere to the workplace, a finding that aligns with the COR theory (Hobfoll, 1989) and the work–home resources model (Wang et al., 2022, 2023). However, it should be emphasised that the cross-sectional design of this study does not allow us to establish the direction of the relationship over time or to infer causality: the findings are consistent with this hypothesis, but do not prove it. Longitudinal replication studies are needed to confirm whether this relationship is indeed directional in the context of CCU.

The explained variance of 15.7% is modest but theoretically coherent for multivariable model in a multidetermined outcome such as occupational burnout. In samples of workers in dual-income couples, the standardised regression coefficients for the effect of parental burnout on work-related burnout typically stand at around β = 0.20 (Wang et al., 2022), whilst studies focusing on strictly occupational factors like workload, lack of role clarity or social support, account for substantially larger proportions of the variance (Mohammadi et al., 2022). The magnitude observed in this study, derived from a sample of CCU professionals under non-pandemic conditions, may represent a estimation of the relationship under normal practice conditions.

Regression analysis using the PBA subscales (F(4, 101) = 4.640, *p* = 0.002, R^2^ = 0.157) revealed that parental emotional distancing was the dimension statistically associated with the BSI (β = 0.409, *p* = 0.006). As an interpretative hypothesis, professionals who have developed a pattern of emotional detachment as a coping response in the parental context may be more likely to employ similar detachment strategies in high-demand professional encounters, in a manner analogous to early forms of occupational depersonalisation (Evans et al., 2023; Mikolajczak & Roskam, 2018). However, given the cross-sectional design and the high intercorrelation between the PBA subscales, which limits the independence of their individual estimates, this interpretation should be treated as a hypothesis for future research, rather than as a confirmed finding. Evans et al. (2023) described how parental burnout impairs executive function and emotional regulation through mechanisms that are not domain-specific; if this pathway is relevant in the context of CCU, it would imply that parental overload could be associated with the professional’s emotional and cognitive availability during their shift. This hypothesis requires direct testing in future studies using a longitudinal design and measuring possible mediating and moderation mechanisms.

### 4.3. Gender Differences in Depersonalisation

A complementary analysis revealed a statistically significant gender difference on the depersonalisation subscale, where male participants scored higher than female participants. This sex-specific pattern was not observed for emotional exhaustion, reduced personal accomplishment, or total burnout, suggesting that depersonalisation in this sample is driven less by parental resource depletion and more by occupational and socially situated factors that differ by sex.

This finding is consistent with the literature, which indicates that men use detachment as a coping strategy more frequently than women, who tend to exhibit exhaustive responses (Cañadas-De la Fuente et al., 2018). Meta-analytic evidence shows a significant positive correlation between male gender and depersonalisation among healthcare professionals (Cañadas-De la Fuente et al., 2018). However, this analysis should be interpreted with caution. Because it was a non-pre-specified exploratory analysis, the male subgroup is small (*n* = 23, 21.7%), and the predominantly female composition of the sample (78.3%) may have lowered the overall mean for depersonalisation, concentrating the risk profile in a small number of men. This finding should be replicated in samples with a more balanced gender distribution before drawing conclusions about a specific male risk profile in CCU.

### 4.4. Perceived Social Support and Flexible Working Hours

As an exploratory analysis, the association between perceived social support (OSSS-3) and flexible working hours and occupational burnout was examined. Perceived social support (OSSS-3) was a significant negatively associated with occupational burnout in the exploratory model (β = −0.211, *p* = 0.032), maintained under bootstrap resampling (*p* = 0.003), and correlated significantly with both EE (ρ = −0.251, *p* = 0.009) and PA (ρ = −0.254, *p* = 0.009). These findings are consistent with COR theory’s proposition that social support constitutes a key environmental resource that buffers against the escalation of resource loss spirals (Hobfoll, 1989), and with empirical evidence in nursing populations (Yeh et al., 2021). It should be emphasised that the 46.2% of participants in the present study reported poor social support (OSSS-3) and only 9.4% reported strong support, a worrying distribution from an occupational health perspective in a context as demanding as that of the CCU.

Perceived flexibility correlated significantly with lower scores on three PBA subscales and with emotional exhaustion but did not emerge as an independent predictor in the multiple regression (β = 0.054, *p* = 0.584). This pattern suggests that flexibility may operate indirectly on occupational burnout by alleviating parental burnout, a mediation pathway consistent with the findings of Maglalang et al. (2021), who demonstrated that scheduling flexibility moderated the combined effect of job and family demands on burnout in healthcare workers. However, this hypothesis needs to be tested using formal mediation models in longitudinal studies with greater psychometric control of this variable in order to be properly verified.

### 4.5. Strengths and Limitations

This study offers several methodological contributions. The multicentre design enhances the representativeness of findings compared to single-institution studies, reducing the risk of institutional idiosyncrasy confounding results. The use of validated, internationally recognised instruments facilitates cross-study comparability. Bootstrap resampling (5000 replications) corroborated all regression findings, enhancing confidence in the primary estimates. However, these strengths should be interpreted alongside several important limitations.

The cross-sectional design precludes causal inference; the directionality of the parental–occupational burnout relationship, while theoretically grounded in the spillover–crossover model and supported by longitudinal evidence, cannot be established from the present data.

Reliance on self-report measures introduces common-method variance, and social desirability bias may have attenuated burnout disclosure—particularly among participants concerned about professional repercussions given the occupational context in which data were collected.

The sample size (*N* = 106) was adequate for the primary bivariate model based on our a priori power calculation. However, because this calculation was designed exclusively for bivariate correlations, a limitation of this study is that Models 1 and 2 lacked dedicated power calculations for multiple regression, meaning these specific multivariable models may be underpowered. Additionally, the sample size constrained statistical power for subgroup analyses (by sex, seniority, and contract type). Findings from these exploratory comparisons should be interpreted cautiously and replicated in larger samples. Also, despite the study targeting all CCU healthcare professionals, no physicians completed the questionnaire. This likely reflects occupational and structural barriers to survey participation among physicians—a pattern documented in healthcare research—rather than an absence of the phenomenon under study. As a result, the sample is effectively limited to nursing staff and allied health workers, and findings cannot be generalised to physicians or to the full interprofessional CCU workforce. In this line, no single-parent participants were identified in the sample (0%). While this may reflect the actual demographic composition of the recruited workforce, it is equally plausible that single parents were systematically less likely to participate due to time constraints or higher baseline burnout, as they face a possible disproportionate parenting burden. This potential self-selection bias may have attenuated estimates of parental burnout severity and its relationship with occupational outcomes.

Participants were recruited exclusively from staff actively working at their posts during the data collection period. Healthcare workers on sick leave, extended medical leave, or in active occupational conflict or burn-out-related absence were therefore not included. Given that the most severely affected individuals are most likely to be on leave, this exclusion introduces a healthy-worker selection bias and is likely to result in conservative estimates of burnout prevalence and effect sizes. Although the multicentre design improves external validity, standard regression analyses do not account for potential non-independence of observations arising from participants being clustered within institutions. Future studies should consider multilevel modelling to partition between- and within-institution variance, particularly where institutional culture and resource availability may moderate the parental–occupational burnout relationship.

The predominantly female sample (78.3%) is broadly representative of the nursing workforce but limits the generalisability of findings—particularly the sex-specific depersonalization finding—to male professionals and non-binary individuals.

An additional psychometric limitation is the relatively low internal consistency observed in certain subscales, particularly PBA-Distancing (ω = 0.658), PBA-Fed-up (ω = 0.652), BSI-EE (ω = 0.638), and BSI-DP (ω = 0.614). Because our major subscale-level regression findings depend heavily on the short three-item PBA-Distancing subscale, these specific dimension-level results must be interpreted cautiously. Future studies utilizing instruments with stronger internal reliability for these specific dimensions are necessary to confirm these findings.

Finally, although found it statistically relevant, the OSSS-3 comprises three conceptually distinct facets of social support, functioning as a composite rather than a unidimensional reflective scale; consequently, internal consistency indices are not the primary validity criterion for this instrument. Nevertheless, the McDonald’s ω obtained in the present sample (ω = 0.584) was below conventionally accepted thresholds, which introduces some imprecision in the operationalisation of perceived social support and should be borne in mind when interpreting the OSSS-3 regression coefficient.

### 4.6. Clinical Implications and Future Directions

The finding that parental burnout explains a significant, if modest, portion of the variance in occupational burnout among CCU healthcare workers carries practical implications for occupational health policy and nursing management. Future longitudinal research should evaluate whether screening for parental burnout. This aspect of personal wellbeing is not typically addressed in occupational health assessments, which are focused exclusively on work-domain variables. This assessment could serve as a useful tool to identify a subgroup of healthcare professionals at elevated risk of occupational burnout prior to its clinical onset. The BR2 framework suggests that targeted interventions addressing the balance between parenting risks and resources (e.g., parenting support programmes, flexible scheduling, peer support groups for parents who are healthcare workers) could reduce parental exhaustion and, through the spillover mechanism, attenuate occupational burnout risk.

The high prevalence of poor perceived social support (46.2%) in this sample identifies an independent target for intervention: evidence-based strategies to strengthen collegial and supervisory social support in CCU environments—such as structured peer support programmes and leadership training in socially supportive management practices—are supported by the existing literature and represent low-cost, scalable organisational interventions.

Future research should prioritise longitudinal designs to establish causal directionality, include larger and gender-balanced samples, and examine the mediating role of emotional regulation and work–family conflict in the parental–occupational burnout pathway. Studies specifically designed to test mediation by work schedule flexibility would address a gap directly suggested by the present correlational pattern.

## 5. Conclusions

This multicentre study indicates a significant association between parental burnout and occupational burnout amongst professionals in critical care units. The results show that parental burnout is a statistically significant predictor of occupational burnout in this setting. Specifically, the dimension of emotional detachment from one’s children carries the greatest weight. At the same time, it was found that greater perceived social support is significantly associated with protection against this burnout.

The findings emphasise that exhaustion arising from the parental role is closely linked to indicators of work-related wellbeing amongst critical care professionals. Therefore, this research provides new insights into how the demands of the family context may be associated with vulnerability in the workplace.

## Figures and Tables

**Table 1:** Sociodemographic and clinical characteristics of participants (*N* = 106).

Characteristic	*n*	%
*Sex*		
Male	23	21.7
Female	83	78.3
*Age group (years)*		
30–40	28	26.4
40–50	56	52.8
>50	22	20.8
*Contract type*		
Permanent	76	71.7
Temporary	30	28.3
*Professional category*		
Registered nurse	65	61.3
Nursing assistant	39	36.8
Healthcare orderly	2	1.9
*Seniority in the unit*		
0–2 years	6	5.7
2–5 years	19	17.9
5–10 years	41	38.7
>10 years	40	37.7
*Perceived social support (OSSS-3)*		
Strong	10	9.4
Moderate	47	44.3
Poor	49	46.2
*Occupational burnout category (BSI)*		
Unlikely	98	92.5
Mild	7	6.6
Moderate	1	0.9

**Table 2:** Descriptive statistics and normality tests for continuous study variables (*N* = 106).

Variable	Median	Mean	SD	IQR	W	*p*
*Occupational Burnout (BSI)*						
Emotional Exhaustion (BSI-EE)	6.00	6.36	3.97	5.75	0.945	<0.001
Depersonalisation (BSI-DP)	2.00	3.07	3.44	4.00	0.822	<0.001
Reduced Personal Accomplishment (BSI-PA)	6.00	6.65	3.67	5.75	0.970	0.017
Total BSI Score	17.00	16.08	8.16	11.75	0.965	0.007
*Parental Burnout (PBA)*						
Parental Emotional Exhaustion (PBA-EE)	13.00	14.83	8.23	13.00	0.950	<0.001
Contrast with previous parent self (PBA-Contrast)	5.00	5.83	6.20	8.00	0.837	<0.001
Feelings of being fed up (PBA-Fed up)	3.00	3.32	2.92	4.00	0.901	<0.001
Emotional distancing from one’s children (PBA-Distancing)	3.00	2.61	2.59	4.00	0.869	<0.001
Total PBA Score	25.00	26.59	17.13	27.75	0.951	<0.001
*Covariate*						
Work Schedule Flexibility	3.00	3.38	1.07	1.00	0.906	<0.001
Social support (OSSS-3)	9.00	8.74	2.05	3.00	0.970	0.017

W = Shapiro–Wilk statistic; SD = standard deviation; IQR = interquartile range.

**Table 3:** Spearman’s Rank correlations among PBA subscales, BSI subscales, and key covariates (*N* = 106).

Variable	1	2	3	4	5	6	7	8	9
1. Number of children	—								
2. Work schedule flexibility	0.081	—							
3. OSSS-3	0.262 **	0.212 *	—						
4. BSI-EE	0.042	−0.193 *	−0.251 **	—					
5. BSI-DP	−0.039	−0.029	−0.001	0.134	—				
6. BSI-PA	−0.076	−0.057	−0.254 **	0.570 ***	0.201 *	—			
7. PBA-EE	−0.070	−0.239 *	−0.094	0.301 **	0.009	0.341 ***	—		
8. PBA-Contrast	0.031	−0.357 ***	−0.115	0.346 ***	0.003	0.312 **	0.621 ***	—	
9. PBA-Fed-up	−0.022	−0.179	−0.078	0.236 *	−0.075	0.222 *	0.785 ***	0.646 ***	—
10. PBA-Distancing	−0.051	−0.235 *	−0.146	0.376 ***	0.080	0.424 ***	0.724 ***	0.700 ***	0.590 ***

* *p* < 0.05; ** *p* < 0.01; *** *p* < 0.001. OSSS-3: Social support; BSI-EE: Emotional Exhaustion; BSI-DP: Depersonalisation; BSI-PA: Reduced Personal Accomplishment; PBA-EE: Parental Emotional Exhaustion; PBA-Contrast: Contrast with previous parent self; PBA-Fed up: Feelings of being fed up; PBA-Distancing: Emotional distancing from one’s children.

**Table 4:** Exploratory multiple linear regression: Predictors of total occupational burnout score (*N* = 106).

Predictor	*B*	SE	β	*t*	*p*	Bootstrap 95% CI	Bootstrap *p*
(Intercept)	19.672	4.627		4.251	<0.001	[11.041, 28.314]	<0.001
Sex (female)	−1.498	1.765		−0.849	0.398	[−4.758, 1.588]	0.321
Perceived flexibility	0.398	0.724	0.054	0.549	0.584	[−1.174, 1.865]	0.607
Social support	−0.816	0.376	−0.211	−2.169	0.032	[−1.489, −0.172]	0.016
Parental burnout	0.136	0.044	0.303	3.098	0.003	[0.062, 0.212]	<0.001
Number of children	−0.312	1.208	−0.025	−0.259	0.797	[−2.475, 2.010]	0.794

Model Summary: R = 0.379, R^2^ = 0.144, Adjusted R^2^ = 0.101, RMSE = 7.292; F(5, 100) = 3.326, *p* = 0.008. *B* = unstandardised regression coefficient; SE = standard error; β = standardised coefficient; CI = confidence intervals.

**Table 5:** Exploratory multiple linear regression: Parental burnout predictors of total occupational burnout score (*N* = 106).

Predictor	*B*	SE	β	*t*	*p*	VIF	Tolerance	Bootstrap 95% CI	Bootstrap *p*
(Intercept)	12.914	1.495		8.640	<0.001			[10.303, 15.906]	<0.001
PBA-EE	−0.010	0.156	−0.010	−0.062	0.951	0.299	3.348	[−0.329, 0.236]	0.888
PBA-Contrast	0.014	0.166	0.011	0.084	0.933	0.470	2.126	[−0.311, 0.317]	0.967
PBA-Fed up	−0.077	0.403	−0.029	−0.190	0.850	0.362	2.760	[−0.873, 0.758]	0.864
PBA-Distancing	1.215	0.432	0.409	2.812	0.006	0.399	2.505	[0.399, 2.091]	0.004

Model Summary: R = 0.396, R^2^ = 0.157, Adjusted R^2^ = 0.123, RMSE = 7.202; F(4, 101) = 4.640, *p* = 0.002. *B* = unstandardised regression coefficient; SE = standard error; β = standardised coefficient; CI = confidence intervals. Collinearity statistics: Variance Inflation Factor (VIF) and Tolerance. PBA-EE: Parental Emotional Exhaustion; PBA-Contrast: Contrast with previous parent self; PBA-Fed up: Feelings of being fed up; PBA-Distancing: Emotional distancing from one’s children.

## Data Availability

The raw data supporting the conclusions of this article will be made available by the authors on request.

## Abbreviations

The following abbreviations are used in this manuscript:

CCU	Critical Care Unit
BSI	Burnout Screening Inventory
BSI-EE	Emotional Exhaustion
BSI-DP	Depersonalisation
BSI-PA	Reduced Personal Accomplishment
PBA	Parental Burnout Assessment
PBA-EE	Parental Role Emotional Exhaustion
PBA-Contrast)	Contrast with Previous Parental Self
PBA-Fed up	Feelings of Being Fed up
PBA-Distancing	Emotional Distancing from One’s Children
